# South African Myxococcota: an untapped resource for microbial ecolo gy and biotechnology

**DOI:** 10.1007/s00253-025-13586-z

**Published:** 2025-08-30

**Authors:** Benjamin Havenga, Karin Jacobs

**Affiliations:** Department of Microbiology, Faculty of Science, Stellenbosch University, Stellenbosch, 7602 South Africa

**Keywords:** Myxococcota, Biosynthetic gene clusters, Antifungal secondary metabolites, Biocontrol agents, Genome mining, South African biomes

## Abstract

**Abstract:**

An extraordinary multicellular life cycle, ecological versatility, and prolific production of bioactive secondary metabolites characterise the phylum Myxococcota. While research has predominantly focused on Myxococcota in Asia, Europe, and North America, their potential occurrence in Sub-Saharan Africa remains largely unexplored. To date, only one study has isolated Myxococcota in South Africa, with additional findings limited to incidental detection through metagenomic studies. Considering South Africa’s ecological diversity, its biomes may represent promising but under-examined environments for systematic bioprospecting aimed at discovering novel Myxococcota with ecological or biotechnological potential. The recent reclassification of Myxococcota from the former Deltaproteobacteria has provided a more coherent taxonomic framework to guide future ecological and systematic studies. This review presents an overview of the taxonomic revision and explores the potential occurrence of Myxococcota in South African biomes. It covers the challenges associated with conventional culture-based isolation methods and highlights potential genome- and metagenome-based approaches, including the use of metagenome-assembled genomes (MAGs) to identify cryptic biosynthetic gene clusters (BGCs), while acknowledging current limitations. Considering the increasing resistance to chemical fungicides in South African agriculture, this review further explores the potential of Myxococcota-derived secondary metabolites as candidate bioprotective alternatives. By identifying current research gaps, it aims to support future efforts towards systematic bioprospecting to investigate the ecological and biotechnological potential of Myxococcota in South Africa.

**Key points:**

• South African biomes may harbour novel Myxococcota with biosynthetic potential.

• Genome mining could reveal cryptic biosynthetic gene clusters (BGCs).

• Myxococcota metabolites may help control resistant fungal phytopathogens.

**Supplementary Information:**

The online version contains supplementary material available at 10.1007/s00253-025-13586-z.

## Introduction

**Fig. 1:**
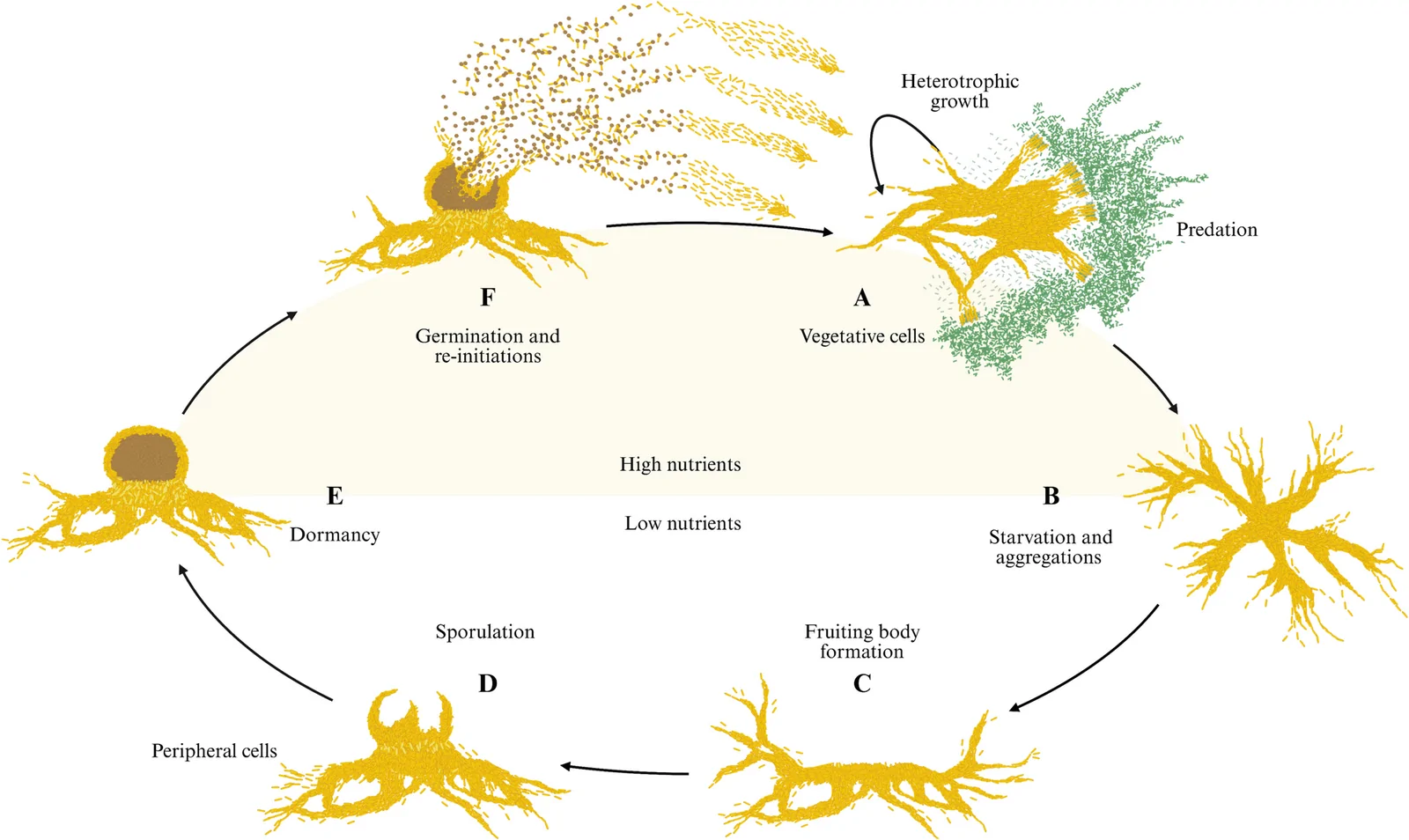
Life cycle of *Myxococcus xanthus* DK1622: Vegetative cells: Under favourable conditions (high nutrients), the rod-shaped *M. xanthus* move *en masse* in coordinated groups (swarming). Cells feed cooperatively to lyse and digest prey (green bacilli) using extracellular enzymes and antimicrobials. This phase reflects heterotrophic growth, where cells consume organic carbon from prey or nutrient media. Starvation and aggregation: Once the cells sense nutrient depletion (low nutrients) or other stress, cells begin signalling each other using cell‐to‐cell contact and chemical cues. Starvation triggers large-scale aggregation as high concentrations of cells converge into aggregation centres. Fruiting body formation: The aggregations mature and form multicellular, dome-shaped fruiting bodies, visually distinct structures where further developmental changes occur. Sporulation: Within the fruiting bodies, cells differentiate into stress-resistant myxospores. Simultaneously, cells lyse (light yellow bacilli) to provide nutrients for sporulation. Peripheral cells refer to non-sporulating cells at the edge of the developing fruiting body, some of which may lyse or remain vegetative. Dormancy: The spores remain dormant in the fruiting body, allowing the population to survive harsh conditions. Germination and re-initiation: As nutrients become available (high nutrients), the spores germinate back into vegetative cells, reinitiate swarming, and the cycle continues. Re-initiation refers to the restart of vegetative growth and swarming following germination (adapted from Mauriello et al. 2010; Dinet et al. 2021; Vos 2021; Created with BioRender.com)

The phylum Myxococcota is characterised by an extraordinary multicellular life cycle (Fig. 1), ecological versatility, and prolific production of biologically active (bioactive) secondary metabolites (Wang et al. 2024). Myxococcota have garnered increasing interest due to their biosynthetic potential, positioning these microorganisms as promising candidates for biotechnological applications. The reclassification of the order Myxococcales into the phylum Myxococcota has provided a more coherent taxonomic framework to support future ecological and systematic studies (Waite et al. 2020; Li et al. 2025). Numerous studies have isolated Myxococcota from terrestrial, aquatic, and extreme environments (Iizuka et al. 1998, 2003; Fudou et al. 2002; Ivanova et al. 2010; Mohr et al. 2017; Tomura et al. 2017; Albataineh & Stevens 2018). However, no systematic studies have been conducted on Myxococcota within South African biomes. To date, only one published report has isolated Myxococcota in South Africa, identifying a bacteriolytic *Myxococcus* sp. from zebra droppings collected in Pietermaritzburg (Memela & Schmidt 2013). Supplementary data from a recent study detected low-abundance Myxococcota taxa in forest soils from the Oribi Gorge Nature Reserve in KwaZulu-Natal (Ogola et al. 2021). Additional metagenomic studies have yielded incidental detections (1 to 2% abundance range), albeit without subsequent culture-based isolation (Matcher et al. 2015; Naidoo 2020; Ogola et al. 2021; Omotayo et al. 2021; Waterworth et al. 2021; Cleary et al. 2023; Botha et al. 2024; León-Sobrino et al. 2024). This review does not summarise an established body of South African Myxococcota research; rather, it highlights the current scarcity of such studies and explores the potential of the different biomes based on global advances and early regional metagenomic findings. Considering South Africa’s ecological diversity, ranging from nutrient-poor Fynbos soils to arid deserts and biologically rich savannas, these biomes may harbour uncharacterised Myxococcota, representing promising environments for future discovery.

The primary challenge in Myxococcota research lies in the difficulty of cultivating these bacteria. Many Myxococcota genera exhibit slow growth and complex interactions within microbial communities, complicating conventional culture-based isolation methods (Dawid 2000; Mohr et al. 2016). Moreover, conventional laboratory conditions are often insufficient to support their growth, as these bacteria rely on specific environmental conditions and growth factors that differ from those of most heterotrophic bacteria (Phillips et al. 2022). Consequently, only a fraction of known Myxococcota have been successfully cultivated, with studies disproportionately focusing on genera such as *Myxococcus* and *Corallococcus* (Groß et al. 2023). However, recent advances in genomics and metagenomics, including metagenome-assembled genomes (MAGs), have facilitated the discovery of cryptic biosynthetic gene clusters (BGCs) that are often inaccessible through conventional culture-based methods (Kautsar et al. 2021; Yue et al. 2023).

Given the challenges posed by fungal phytopathogens in South Africa, Myxococcota could potentially represent a source of promising bioprotective solutions. International studies have demonstrated the antifungal potential of Myxococcota-derived metabolites (Dahm et al. 2015; Wu et al. 2021; Xia et al. 2023), although their application in South African agricultural systems remains largely unexplored. Fungal diseases pose a significant threat to South African agriculture, with pathogens such as *Botrytis*, *Alternaria*, and *Fusarium* spp. resulting in notable yield losses (Janse van Rensburg et al. 2016; Benjamin et al. 2024). Moreover, the widespread use of chemical fungicides contributes to the emergence of resistant fungal populations, reducing the efficacy of conventional chemical fungicides, while raising concerns related to environmental impact and food security (Wroński et al. 2024; Lazarević-Pašti et al. 2025). In response to these challenges, Myxococcota-derived secondary metabolites may offer alternative antifungal strategies to chemical fungicides, as they have been reported to exhibit diverse mechanisms of action and bioactivities against phytopathogens in global studies (Gerth et al. 1980, 1996a; Wu et al. 2021; Xia et al. 2023; Yue et al. 2023; Han et al. 2024, 2025). Despite the progress of international studies, Myxococcota-based biocontrol strategies have not yet been evaluated within a South African context, and their broader agricultural potential remains underexplored (Dahm et al. 2015; Wu et al. 2021; Xia et al. 2023).

This review synthesises research on Myxococcota in relation to South African biomes. It outlines the recent taxonomic reclassification, assesses available evidence for their biome-specific distribution, and discusses ongoing challenges associated with conventional culture-based isolation and cultivation. Genomic- and metagenomic-based strategies, including the use of MAGs to uncover cryptic BGCs, along with their current limitations in assembly quality and functional prediction, are discussed. Considering the rising fungicide resistance in South African agriculture, the review also covers the potential of Myxococcota-derived secondary metabolites as bioprotective alternatives. By identifying current research gaps, it aims to support future bioprospecting efforts to investigate the ecological role and biotechnological potential of Myxococcota in South Africa.

## Taxonomic reclassification of Myxococcota

**Fig. 2:**
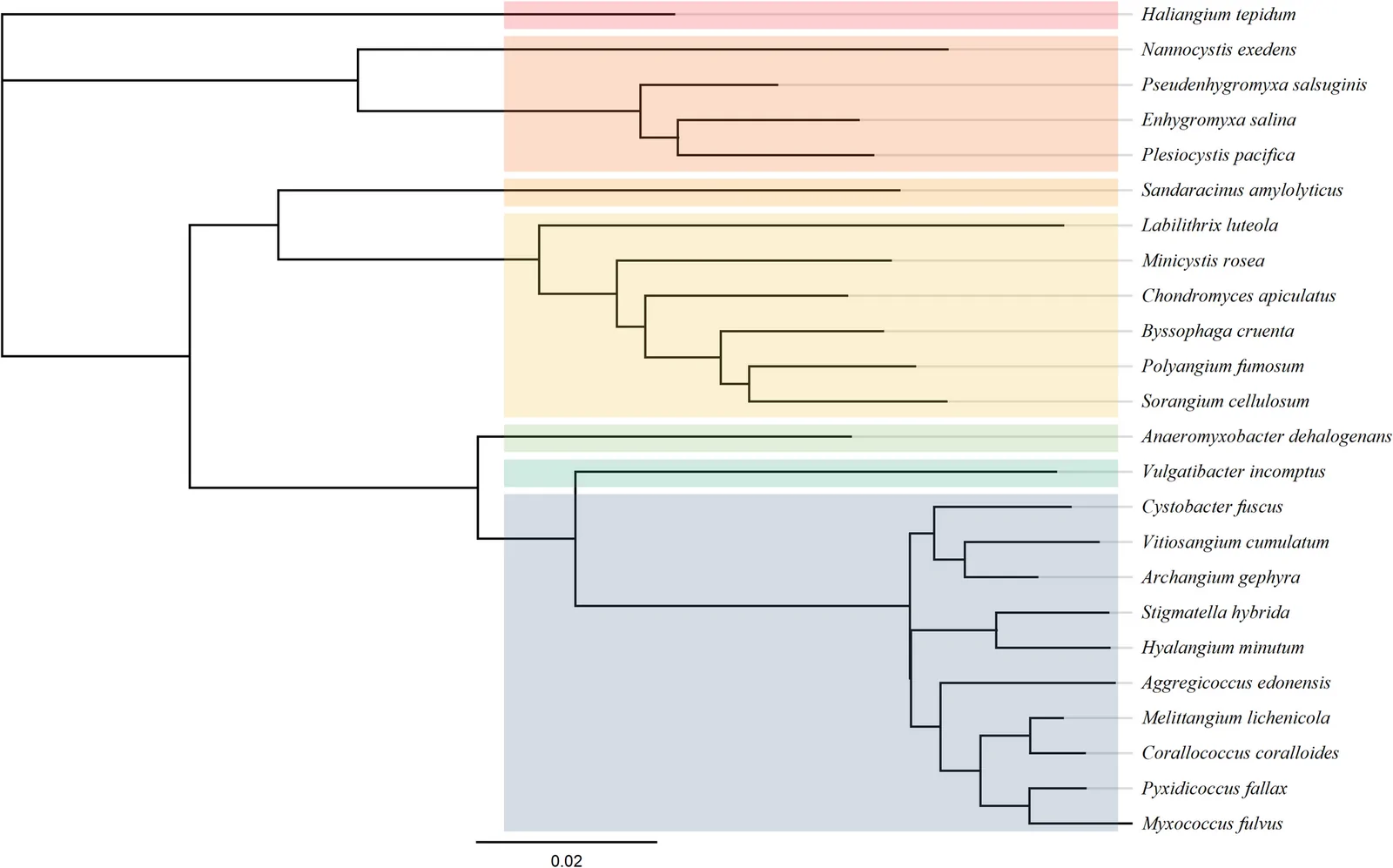
Phylogenetic tree of Myxococcota strains based on 16S rRNA gene sequences. Selected type strains representing the major families were obtained from the DSMZ database (https://www.dsmz.de/), and their 16S rRNA gene sequences were retrieved from NCBI GenBank (https://www.ncbi.nlm.nih.gov/). Sequences were aligned using MUSCLE v5.1 (Geneious Prime® 2025.2.1, default settings). The phylogenetic tree was constructed using the Neighbour-Joining method with the Tamura-Nei genetic distance model. No outgroup was applied. Bootstrap analysis was performed with 100 replicates, and bootstrap values ≥ 50% are indicated at the nodes. Strains are colour coded by family (from top to bottom): Haliangiaceae (red), Nannocystaceae (peach), Sandaracinaceae (orange), Polyangiaceae (yellow), Anaeromyxobacteraceae (green), Vulgatibacteraceae (teal), and Myxococcaceae (grey blue). Scale bar (0.02) represents the number of nucleotide substitutions per site, indicating genetic distance. Corresponding 16S rRNA gene sequences are available in the Supplementary File 1 (adapted from Waite et al. 2020 and Whitworth et al. 2021)

Previously, myxobacteria (a non-taxonomic general term) were classified as the order Myxococcales within the class Deltaproteobacteria, containing three suborders (Cystobacterineae, Nannocystineae and Sorangiineae) and eight families (Myxococcaceae, Archangiaceae, Vulgatibacteraceae, Phaselicystidaceae, Polyangiaceae, Sandaracinaceae, Nannocystaceae, and Haliangiaceae) (Li et al. 2025). More recently, Waite et al. (2020) conducted a MAG-based analysis of the Deltaproteobacteria class, which led to the proposed reclassification of the former order Myxococcales. The genus *Phaselicystis* within the family Phaselicystidaceae was integrated into the synonymous Polyangiaceae family. Similarly, the reclassification merged Archangiaceae with the Myxococcaceae family, except for the genus *Anaeromyxobacter*, for which a separate family, Anaeromyxobacteraceae, was designated (Waite et al. 2020; Whitworth et al. 2021). Overall, the former order Myxococcales was reclassified under the newly established phylum Myxococcota, comprising two classes (Myxococcia and Polyangia), four orders (Myxococcia: Myxococcales; and Polyangia: Polyangiales, Nannocystales, and Haliangiales), and seven families (Fig. 2) (Waite et al. 2020).

The reclassification offers improved phylogenetic resolution and provides a more coherent framework to support future ecological, systematic, and bioprospecting studies (Waite et al. 2020). With the updated framework in place, South Africa’s ecologically diverse biomes may represent an untapped reservoir for systematic bioprospecting studies aimed at discovering novel Myxococcota with ecological or biotechnological potential.

## South African biomes as promising but underexplored reservoirs of Myxococcota

### Overview of South Africa’s biomes and their microbial potential

Historically, Myxococcota have been described as inhabitants of terrestrial ecosystems, primarily soil. However, advancements in sequencing technologies have revealed their presence across a broader range of terrestrial and aquatic environments (Dawid 2000; Mohr 2018; Wang et al. 2021). These include acidic moors and fens, sandy island soil and compost, subterranean subsurface, and caves (Brinkhoff et al. 2012; Mohr et al. 2016; Bhat et al. 2021; Dai et al. 2024). Culture-based methods have isolated species such as *Myxococcus*, *Corallococcus*, and *Archangium* from mangroves, marine systems, saline soils, deserts, hot springs, and Arctic environments (Iizuka et al. 1998; Jiang et al. 2010; Li et al. 2012). They have also been isolated from decaying plant matter and herbivore dung, including zebra droppings from South Africa (Reichenbach 1999; Memela & Schmidt 2013; Sharma et al. 2022) (Table 1). These findings suggest the ecological versatility of Myxococcota and raise a central question: do South Africa’s biomes harbour novel, yet undiscovered members of this phylum?

**Table 1:** Summary of Myxococcota diversity reported from various countries and biomes, indicating sample type, detected families, and whether strains were isolated

Country	Biome	Sample type	Family^†^	Isolated^‡^	Reference
Antarctica	Antarctic polar desert	Soil	Unclassified Myxococcota	No	Benaud et al. (2022)
Austria	Temperate forest	Forest litter/soil	Haliangiaceae	No	Petters et al. (2021)
			Polyangiaceae		
			Nannocystaceae		
Czech Republic	Floodplain	Soil	Haliangiaceae		
			Polyangiaceae		
Norway	Wetland	Peatland soil	Haliangiaceae		
			Polyangiaceae		
Spain	Shrubland	Soil	Myxococcaceae		
			Haliangiaceae		
			Polyangiaceae		
UK/Germany	Grassland	Soil	Haliangiaceae		
			Polyangiaceae		
Canada	Boreal forest	Rhizosphere soil	Unclassified Myxococcota	No	Chow et al. (2002)
China	Freshwater	Sediment	Myxococcaceae	No	Li et al. (2012)
			Polyangiaceae		
			Nannocystaceae		
Colombia	Tropical rainforest	Soil and forest litter	Anaeromyxobacteraceae	No	Gómez et al. (2024)
Germany	Wetland	Soil, water sample and faeces	Myxococcaceae	Yes	Mohr et al. (2016)
			Polyangiaceae		
			Nannocystaceae		
Germany/Norway	Marine/coastal	Seawater and sediment	Myxococcaceae	Yes	Brinkhoff et al. (2012)
Greenland	Arctic tundra	Permafrost soil	Unclassified Myxococcota	No	Scheel et al. (2023)
India	Agricultural	Sheep and goat dung	Myxococcaceae	Yes	Sharma et al. (2022)
	Temperate broadleaf forest				
	Semi-arid/arid				
	Temperate forest				
Indonesia	Mangrove	Sediment and leaf flakes	Myxococcaceae	Yes	Octaviana et al. (2023)
			Polyangiaceae		
			Nannocystaceae		
Japan	Marine/coastal	Sand and seaweed	Nannocystaceae	Yes	Iizuka et al. (1998)
		Water, sand, gypsum precipitate, biomat, mud, fallen leaf, sulphur precipitate and wood piece	Polyangiaceae		
			Haliangiaceae		
			Nannocystaceae	Yes	Iizuka et al. (2006)
Mexico	Semi-arid/arid	Soil	Myxococcaceae	Yes	Brockman (1976)
			Polyangiaceae		
Namibia	Semi-arid/arid	Soil	Unclassified Myxococcota	No	Naidoo (2020)
Norway	Arctic tundra	Permafrost soil	Myxococcaceae	No	Schostag et al. (2019)
			Haliangiaceae		
			Nannocystaceae		
			Polyangiaceae		
			Sandaracinaceae		
South Africa	Savanna	Zebra droppings	Myxococcaceae	Yes	Memela & Schmidt (2013)
	Marine/coastal	Estuarine water	Nannocystaceae	No	Matcher et al. (2015)
			Polyangiaceae		
		Estuarine sediment	Myxococcaceae		
			Nannocystaceae		
			Polyangiaceae		
		Seawater	Myxococcaceae	No	Waterworth et al. (2021)
			Unclassified Myxococcota	No	Cleary et al. (2023)
	Forest	Soil	Myxococcaceae	No	Ogola et al. (2021)
	Grassland		Unclassified Myxococcota	No	Omotayo et al. (2021)
	Semi-arid/arid		Unclassified Myxococcota	No	Baluka (2023)
	Savanna		Unclassified Myxococcota	No	Botha et al. (2024)
USA	Temperate broadleaf forest	Soil	Myxococcaceae	Yes	Ahearne (2024)
			Polyangiaceae		
			Nannocystaceae		

^†^Unclassified Myxococcota—sequences assigned only at the phylum level (not resolved family classification)

^‡^Isolated—Yes: indicates that Myxococcota strains were successfully isolated; No: indicates detection through molecular or metagenomic methods only. Family assignments follow the revised taxonomy of Waite et al. (2020) and Whitworth et al. (2021)

**Fig. 3:**
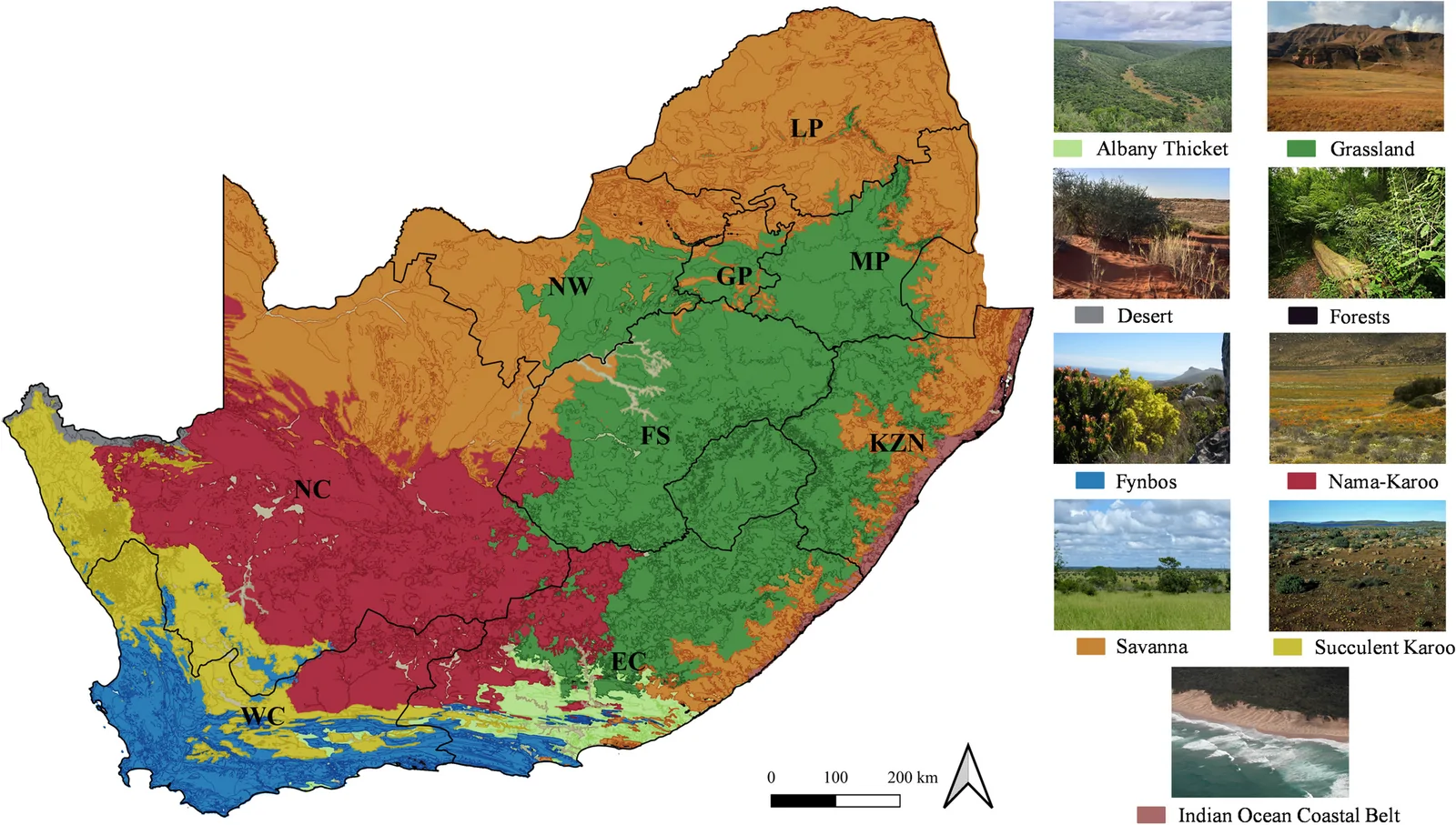
Map of South Africa’s biomes based on the Terrestrial Ecosystem National Vegetation Map 2024 dataset. The biomes are categorised based on dominant vegetation and ecological function. Data was sourced from the South African National Biodiversity Institute (2006–2024) and processed using the QGIS software 3.32 (https://qgis.org/). Biome photos were adapted from freely licensed Wikimedia Commons (https://commons.wikimedia.org/wiki/Main_Page) and used for illustrative purposes in accordance with their respective Creative Commons licenses (full photo credits and source links are listed in the Supplementary File 3). The map includes a north arrow and a scale bar (bottom right) indicating spatial reference (0 to 200 km). Province abbreviations: EC – Eastern Cape; FS – Free State; GP – Gauteng; KZN – KwaZulu-Natal; LP – Limpopo; MP – Mpumalanga; NC – Northern Cape; NW – North West; WC – Western Cape

Ecologists divide South Africa into nine biomes: Fynbos, Succulent Karoo, Desert, Nama-Karoo, Grassland, Savanna, Albany Thicket, Indian Ocean Coastal Belt, and Forests (Fig. 3) (Sell et al. 2024). These regions encompass a wide range of environmental conditions that may be conducive to Myxococcota; however, their distribution remains largely uncharacterised. Considering the country’s ecological diversity, these biomes may represent promising reservoirs of unexplored Myxococcota with biotechnological relevance. Given their metabolic adaptability and dual roles as microbial predators and decomposers, Myxococcota may contribute significantly to microbial community dynamics within these environments, despite the scarcity of current research.

#### Fynbos biome

The Cape Floristic Region (CFR) is characterised by exceptional floral diversity, nutrient-poor soils, and winter rainfall patterns. The CFR encompasses diverse vegetation types, broadly summarised as Sand Fynbos, Alluvium Fynbos, Granite Fynbos, Sandstone Fynbos, Shale Fynbos, Renosterveld, Cape Flats Dune Strandveld, Cape Seashore Vegetation, and the Southern Afro-temperate Forest (Fig. 3) (Rebelo et al. 2006; Jacobs et al. 2020; Sell et al. 2024). Fynbos biomes are characterised by fine-leaved fire-adapted shrubs that require periodic burns (every 10 to 15 years) for regeneration (Sell et al. 2024; Vermonti et al. 2025). Compared to other biomes where herbivores significantly influence nutrient cycling, Fynbos ecosystems experience minimal grazing pressure due to the low palatability of plants and poor soil fertility. Microbial communities, therefore, play a central role in nutrient cycling (Bengtsson et al. 2011).

Amongst these microbial communities, Myxococcota are well known for their ability to degrade complex organic substrates (cellulose, lignin, pectin, and chitin) as well as recalcitrant carbon sources found in shrubs and fire-affected plant material (Carr et al. 2013; Saraf and Sharma 2024). Therefore, the Fynbos biome may offer a suitable habitat for Myxococcota, including *Sorangium* and *Chondromyces*, which grow in habitats with slow decomposition rates (Bode and Müller 2007). Additionally, *Polyangium*, frequently found in nutrient-poor or oligotrophic soils, may also be present in the Fynbos biome, where predator–prey interactions and recalcitrant organic matter decomposition are key for survival (Reichenbach 1999; Mohr 2018; Finn et al. 2021; Petters et al. 2021; Li et al. 2023). These ecological factors suggest that Myxococcota could form part of an underexplored microbial community, facilitating nutrient availability in Fynbos soil. Although some species have been isolated from comparable oligotrophic habitats globally, they have been infrequently reported in Fynbos-specific microbiome studies (Slabbert et al. 2014; Postma et al. 2016). Their low detectability may result from methodological constraints or seasonal fluctuations. Clarifying the carbon source preference of Myxococcota in Fynbos soil could offer insights into their specific ecological roles within the biome. While many Myxococcota favour complex organic substrates, it remains unclear whether they specialise in degrading recalcitrant sources derived from Fynbos vegetation (Murphy et al. 2021; Gladkov et al. 2022; Xu et al. 2024).

Additionally, as Fynbos is fire-dependent, fire events contribute substantially to nutrient cycling by generating rapid pulses of nutrients through ash decomposition, which are rapidly depleted due to leaching and microbial uptake (Bergh & Compton 2015; Verboom et al. 2024). Ash deposition also influences microbial community dynamics, favouring microorganisms that facilitate pyrogenic carbon decomposition and secondary carbon metabolism (Pellegrini et al. 2021). Although no systematic studies have assessed South African Myxococcota post-fire, population shifts may occur following fire events. Such shifts could be influenced by increased inorganic nitrogen levels and altered carbon availability resulting from organic matter volatilisation (Ding et al. 2024; Qi et al. 2024). Concomitantly, these factors highlight the potential role of Myxococcota as underexplored but ecologically relevant members of the Fynbos microbiome, contributing to carbon cycling, recalcitrant substrate breakdown, and post-fire nutrient dynamics in this endemic, oligotrophic environment.

#### Arid and semi-arid biomes

The Succulent Karoo, Nama-Karoo, and Desert biomes collectively represent South Africa’s arid regions, with low rainfall, temperature variability, and nutrient-poor soils (Fig. 3) (Arena et al. 2018). These biomes host slow-decomposing shrubs, ephemeral plants, and unique perennials including *Welwitschia mirabilis* (Carr et al. 2013; Valverde et al. 2016; Arena et al. 2018; Venter et al. 2021; Doniger et al. 2022; Sell et al. 2024). Although Myxococcota have not been directly isolated from these biomes in South Africa, indirect metagenomic and transcriptomic data from comparable Sub-Saharan environments suggest their presence at low abundance (Naidoo 2020; León-Sobrino et al. 2024). Factors such as nutrient limitations, sporadic rainfall, and predator–prey dynamics may constrain their abundance or favour transient or dormant populations (Cowan et al. 2023; Richy et al. 2024). Slow decomposition may lead to accumulated recalcitrant carbon sources, creating an environment for Myxococcota capable of degrading complex organic matter (Zhao et al. 2024; Peng et al. 2025). Nitrogen limitation may promote organic nitrogen acquisition in Myxococcota (non-nitrogen-fixing organisms) via facultative predation on lysed prey (Bauer and Forchhammer 2021). Furthermore, the potential interaction between Myxococcota and diazotrophic bacteria could provide an indirect nitrogen source, enabling their survival and persistence in nutrient-poor environments (Acinas et al. 2021). Moreover, Myxococcota may also persist under arid conditions through dormancy mechanisms, such as the formation of fruiting bodies and spores (myxospores) (Lall et al. 2024). These adaptations suggest that arid biomes may harbour Myxococcota, such as *Polyangium*, *Chondromyces*, and possibly *Sorangium* (Bode & Müller 2007; Mohr et al. 2017; Mohr 2018; Finn et al. 2021; Lall et al. 2024).

The Grassland biome is the second largest in South Africa, characterised by the absence of trees and shrubs and the presence of grasses (hemicryptophytes) (Fig. 3). Two grassland classes are recognised: sour Grasslands in moist, leached soils with low palatability, and sweet Grasslands in drier, nutrient-richer soils (Sell et al. 2024). Fire and herbivory play essential roles in maintaining the grasslands, which prevent forest formation in high-rainfall areas (Sell et al. 2024). Fire events drive nutrient pulses via ash decomposition, altering soil microbial communities by increasing microbial biomass turnover, which may favour the transient activity of Myxococcota (Bergh & Compton 2015; Pellegrini et al. 2021; Chen et al. 2023; Verboom et al. 2024). The absence of trees and shrubs alters rhizodeposit profiles and increases grass-derived organic inputs, potentially supporting Myxococcota adapted to high microbial turnover and shallow rhizospheres (Urbanová et al. 2011; Prommer et al. 2020; Vaupel et al. 2025). These conditions may support genera such as *Myxococcus*, *Sorangium*, and *Polyangium*, which are associated with detritus turnover and microbial predation (Bill 2019; Stoner et al. 2021).

Human-driven grassland transformation through cropping and timber plantations (Sell et al. 2024), along with agricultural inputs like tilling and fertilisers, alters microbial dynamics, enhancing biomass turnover and predator–prey interactions (Dai et al. 2021; Górska et al. 2022). Myxococcota may act as saprophytic or predatory agents in these environments. Supporting this, Omotayo et al. (2021) detected Myxococcota in maize rhizospheres from Lichtenburg and Randfontein in South Africa using metagenomic methods. These findings suggest that grassland soils, particularly under anthropogenic influence, may represent promising but underexplored reservoirs of agriculturally relevant Myxococcota.

#### Savanna biome

The Savanna biome, one of the largest biomes in southern Africa, is characterised by perennial trees, shrubs, grasses, and well-drained loamy soils. Fire, herbivory, and climatic variability influence its nutrient dynamics (Fig. 3) (Sell et al. 2024). Metagenomic studies have detected Myxococcota in savanna soils, including beneath *Acacia* canopies and across biological soil crusts, suggesting their role in organic matter decomposition and nutrient cycling (Baluka 2023; Botha et al. 2024). Vegetation structure influences microbial diversity via nitrogen fixation and root exudates, potentially supporting Myxococcota abundance (Duan 2022; Alharbi et al. 2025). Their role in carbon sequestration is also plausible, through the decomposition of rhizodeposits and lignin-rich litter, by genera such as *Corallococcus* and *Sorangium* (Reichenbach 1999; Urbanová et al. 2011; Prommer et al. 2020; Petters et al. 2021; Vaupel et al. 2025).

Similar to other biomes, fire events create transient nutrient pulses that may favour resilient Myxococcota, including *Myxococcus* and *Sorangium*, which utilise newly available organic substrates (Ding et al. 2024; Lall et al. 2024). Herbivore dung, a microbial hotspot, may support genera such as *Myxococcus*, *Cystobacter*, *Stigmatella*, and *Polyangium*, which thrive in nutrient-rich, prey-dense environments (Sharma et al. 2022; Ahearne 2024; Saraf and Sharma 2024). Additionally, burned dung may offer distinct micronutrient niches, enriched in calcium, phosphorus, iron, and cobalt, potentially favouring Myxococcota adapted to such conditions (Mohr 2018; Sánchez-García et al. 2025).

#### Marine and coastal biomes

The Albany Thicket and Indian Ocean Coastal Belt biomes exhibit diverse vegetation and sandy, fertile soils, sharing ecological characteristics with the Savanna and Grassland biomes (Fig. 3) (Sell et al. 2024). Myxococcota have been detected in South African estuarine and marine environments. Metagenomic studies have confirmed the presence of Myxococcota in estuaries (Matcher et al. 2015), *Latrunculia* sponges from protected coastal regions (Waterworth et al. 2021), and calcareous sponges near Rodrigues Island (Cleary et al. 2023). These findings suggest that Myxococcota may play roles in carbon, nitrogen, and phosphorus cycling within estuarine microbial communities (Murphy et al. 2021; Rodríguez-Ramos et al. 2022; Fan et al. 2023; Peng et al. 2024; Zou et al. 2024).

Within these environments, Myxococcota may degrade detritus and sponge exudates, influence nitrogen availability in anaerobic sediments, and contribute to phosphorus solubilisation, supporting microbial and plant growth (Waterworth et al. 2021; Zou et al. 2024). Genera including *Myxococcus*, *Corallococcus*, *Archangium*, *Chondromyces*, and *Nannocystis* may be involved in coastal carbon cycling (Octaviana et al. 2023). A few marine Myxococcota have been reported to tolerate high salinity via osmolyte accumulation, including species such as *Haliangium ochraceum*, *Plesiocystis pacifica*, and *Enhygromyxa salina* (Fudou et al. 2002; Jiang et al. 2010; Tomura et al. 2017). Sponge-associated and estuarine habitats may thus represent reservoirs of unique, biotechnologically promising Myxococcota (Da Costa et al. 2023; Skoog et al. 2023).

#### Forest biome

The forest biome in southern Africa include Afrotemperate forests as well as tropical forests often embedded in other biomes, typically fire refugia (Sell et al. 2024). Although direct Myxococcota research in African Forest biomes remains limited, Ogola et al. (2021) detected Archangiaceae in soils collected from the Oribi Gorge Nature Reserve in KwaZulu-Natal. Forest ecosystems are rich in decaying plant matter, leaf litter, and humus, providing carbon and nitrogen-rich substrates that support microbial activity. These conditions, often complemented by nitrogen-fixing microorganisms, may support Myxococcota populations (Urbanová et al. 2015; Murphy et al. 2021; Petters et al. 2021; Peng et al. 2024; Shi et al. 2024; Richy et al. 2024; Zhang et al. 2024a, b). Genera such as *Haliangium*, *Polyangium*, *Myxococcus*, and *Corallococcus* have been associated with decaying debris, while *Archangium* and *Chondromyces* have been observed in rhizosphere niches (Reichenbach 1999; Dawid 2000; Mohr et al. 2016; Sasse et al. 2018; Petters et al. 2021).

### Biogeographical relevance and research gaps

One published study by Memela and Schmidt (2013) directly investigated Myxococcota in Pietermaritzburg (KwaZulu-Natal) (Fig. 3), providing the only targeted analysis to date in South Africa. In contrast, all other studies have identified Myxococcota incidentally, as part of broader metagenomic analyses of microbial communities (Matcher et al. 2015; Naidoo 2020; Ogola et al. 2021; Omotayo et al. 2021; Waterworth et al. 2021; Cleary et al. 2023; Botha et al. 2024; León-Sobrino et al. 2024). Considering South Africa’s ecological diversity, there is a clear need for systematic isolation studies across its major biomes to comprehensively characterise Myxococcota diversity, ecological function, and potential biotechnological value.

## Isolation, cultivation challenges, and genomic approaches

Metagenomic studies have offered insight into the presence and potential diversity of Myxococcota in various biomes, suggesting that cultivation has so far recovered only a fraction of the Myxococcota. This primarily results from the implementation of conventional culture-based isolation methods, which the “great plate count anomaly” continues to challenge (Staley & Konopka 1985). Myxococcota are characterised by slow and complex growth, predation, and the requirement for complex interactions with other microbial communities, making in vitro laboratory cultivation challenging. Conventional culture–based isolation methods often fail to recover a significant fraction of Myxococcota diversity, as many species require specific environmental conditions and the presence of various growth factors (Mohr et al. 2016; Lewis et al. 2021). Research has largely focused on the genera *Myxococcus* and *Corallococcus* due to their ease of cultivation, bioactivity, and availability of genome sequences (Mohr 2018; Whitworth et al. 2020; Wang et al. 2024). At the same time, researchers have not cultivated many Myxococcota in vitro, and their ecological role remains elusive (Groß et al. 2023).

### Conventional culture–based isolation

Environmental conditions (temperature, pH, and oxygen concentration) and growth factors (vitamins, amino acids, nucleotides, inorganic compounds, humic acid, or other electron shuttles) represent significant challenges in cultivating Myxococcota (Lewis et al. 2021; Li et al. 2025). Functionally, they have been broadly grouped into two overlapping groups: Group I (cellulolytic) and Group II (bacteriolytic) (Dawid 2000). Cellulolytic Myxococcota utilise inorganic nitrogen when grown on cellulose or glucose but are suppressed under high sugar concentrations, while bacteriolytic (Group II) Myxococcota depend on amino acid-containing substrates derived from the enzymatic degradation of proteins (Dawid 2000; Phillips et al. 2022). Recent studies, however, suggest this dichotomy is oversimplified, as many Myxococcota display metabolic flexibility and may switch between strategies depending on environmental conditions (Phillips et al. 2022; Ahearne 2024). Moreover, recently isolated species, such as *Vulgatibacter incomptus*, *Labilithrix luteola*, and *Simulacricoccus ruber*, do not fit either category, suggesting alternative modes of nutrient acquisition (Yamamoto et al. 2014; Garcia and Müller 2018). This functional complexity complicates efforts to standardise isolation procedures, as many Myxococcota appear to have highly specific and variable growth requirements.

Conventional culture–based isolation methods often involve using low-nutrient agar (water agar), combined with baiting using dung, live or dead prey (bacteria, yeast, or filamentous fungi), or cellulose filter paper (Reichenbach 1983; Dawid 2000). However, these isolation methods face challenges as Myxococcota exhibit complex collective behaviour, including swarming and multicellular fruiting body development (Fig. 1) (Faiza et al. 2025). These behaviours require solid surfaces and specific humidity levels, which are often difficult to recreate in vitro (Muñoz-Dorado et al. 2016; Whitworth et al. 2021; Zhang et al. 2023). Compounding the difficulty, other microorganisms (prokaryotic or eukaryotic) outgrow and outcompete Myxococcota. Moreover, Myxococcota are sensitive to antimicrobials (erythromycin, neomycin, kanamycin, streptomycin, tetracycline, and actinomycin), limiting the effectiveness of antimicrobial selection during isolation (Dawid 2000; Mohr et al. 2016).

To overcome these limitations, modified techniques have been developed to improve enrichment and reduce contamination, including freezing, heating, sonication, and treatment with antifungal (cycloheximide, amphotericin, natamycin, and nystatin), antibiotic (chloramphenicol), or crystal violet (Saggu et al. 2023). Additionally, several innovative technologies (based on or expanded from conventional culture–based isolation methods) may become applicable in the future, including the isolation chip (iChip), hollow-fibre membrane chambers, diffusion bioreactors, or the soil substrate membrane system (Lewis et al. 2021). While these methods may support Myxococcota isolation from various environmental sources, additional factors may require optimisation, including bait selection (dung, bacteria, yeast, filamentous fungi, or cellulose filter paper), medium composition (physicochemical properties), cultivation temperature and duration, and chemical treatment (antibiotic, antifungal or crystal violet) (Lewis et al. 2021; Li et al. 2025).

### Genomics-based approaches to unlock cultivation challenges

Despite continued efforts to improve conventional culture–based isolation methods for Myxococcota, cultivation challenges have limited access to BGCs, due to their silent nature under conventional laboratory conditions. These silent or non-expressed BGCs are commonly referred to as cryptic BGCs, as their metabolic products remain unknown until they are expressed under specific environmental conditions or in the presence of growth factors (Kautsar et al. 2021; Panter et al. 2022). The integration of bioinformatic tools and databases has facilitated genome mining, enabling researchers to identify cryptic BGCs in Myxococcota (Sourice et al. 2024; Wang et al. 2024). Rather than relying solely on conventional culture-based isolation methods, advanced genomics and post-genomic techniques have enabled the identification and prediction of novel BGCs, paving the way for future bioprospecting and expression studies (Garcia and Müller 2020; Whitworth et al. 2021).

### Genomic approaches that bypass cultivation challenges

Several databases and bioinformatic tools are available for use and may allow the prediction and categorisation of Myxococcota BGCs (Sourice et al. 2024; Wang et al. 2024). Databases and bioinformatic tools include Antibiotics and Secondary Metabolite Analysis Shell (antiSMASH), antiSMASH-DB, Integrated Microbial Genomes–Atlas of Biosynthetic gene Clusters (IMG–ABC), Minimum Information about a Biosynthetic Gene Cluster (MIBiG), Biosynthetic Gene Similarity Clustering and Prospecting Engine (BiG-SCAPE), and Biosynthetic Gene cluster Families database (BiG-FAM) (Hadjithomas et al. 2015; Blin et al. 2019; Navarro-Muñoz et al. 2020; Kautsar et al. 2021). antiSMASH, for example, detects biosynthetic pathways by comparing genetic sequences to known BGCs, while IMG-ABC enables functional annotation of microbial secondary metabolites (Blin et al. 2021). MIBiG and BiG-FAM group BGCs based on structural similarities, aiding comparative genomics and the classification of biosynthetic families across bacterial taxa (Kautsar et al. 2020; Kautsar et al. 2021; Panter et al. 2022).

As many Myxococcota remain unculturable, metagenomic approaches, such as MAGs, may allow for the assessment of the biosynthetic potential of bacteria without the need for culturing. MAGs enable the reconstruction of microbial communities directly from environmental samples, allowing for the potential identification of previously cryptic BGCs (Whitworth et al. 2021; Li et al. 2023). In one example, Kurashita et al. (2024) used shotgun metagenomics to reconstruct 46 Myxococcota MAGs and predicted a total of 533 biosynthetic gene clusters (BGCs) using antiSMASH. The authors further used NaPDoS (The Natural Product Domain Seeker) to identify 373 ketosynthase (KS) domains from PKSs and 66 condensation (C) domains from NRPSs, suggesting a high potential for diverse secondary metabolite production.

While MAGs offer a comprehensive bioinformatic approach for identifying BGCs, several limitations must be considered when interpreting results. For example, MAGs that are assembled from short-read sequence data are often fragmented. BGC regions that code for modular enzymes [polyketide synthases (PKSs) or non-ribosomal peptide synthases (NRPSs)] are characterised by highly repetitive DNA coding regions. These regions are difficult to accurately assemble and can result in incomplete BGC prediction (Sánchez-Navarro et al. 2022). Another important consideration is the selection of sequencing technology, which influences the quality of genome assembly and MAGs. Short-read platforms (Illumina MiSeq) allow for the sequencing of 100 to 300 base pairs; however, struggle to cover longer repetitive regions common in BGCs. In contrast, long-read sequencing platforms [Pacific Biosciences (PacBio) and Oxford Nanopore Technologies (ONT)] facilitate the generation of long DNA fragments (10 to 12 kilobases) allowing for a more complete assembly of full-length BGCs. However, it is important to note that long-read platforms have a higher per-read error rate compared to short-read counterparts. Therefore, a hybrid approach encompassing both short- and long-read platforms could be considered, enhancing both accuracy and genome completeness, resulting in higher-quality MAGs (Mirete et al. 2025).

Additionally, while several bioinformatic tools have allowed for the identification of BGCs, the functional annotation of the encoded enzymes and prediction of the resulting metabolites still present a significant challenge. As genome mining relies on sequences of known homologous enzymes or explicitly defined criteria for identifying BGCs, results are limited to the availability of already characterised biosynthetic types and families in databases (Ziemert et al. 2016). Zhang et al. (2024a, b) conducted a large-scale metagenomic analysis of soil-derived BGCs and found that 38.1% of gene cluster families showed no overlap with BGCs in BiG-FAM, while 59.7% were not represented in MIBiG. These findings highlight the limitations of currently available databases, underrepresenting unculturable taxa in the environment. While in silico approaches provide insight into the potential diversity of BGCs, they should be complemented with in vitro assays to confirm novel BGCs and secondary metabolites (Zhang et al. 2024a, b). One potential option involves heterologous expression of the novel BGCs. However, activating and expressing these clusters remain challenging, requiring further research into optimising regulatory mechanisms, expression systems, and host compatibility (Whitworth et al. 2021; Yue et al. 2023; Kurashita et al. 2024). Additionally, heterologous expression of BGCs remains a bottleneck, as no MAG-derived Myxococcota BGCs have been fully expressed and validated in hosts (Yue et al. 2023). Although early secondary metabolites such as epothilones, chondramides, and myxovalargin were discovered prior to the genomic era, genome mining has since clarified their biosynthetic origins and predicted many additional, uncharacterised BGCs.

These BGCs and the accompanying secondary metabolites are being explored for their potential application in agriculture, as biocontrol agents against fungal pathogens. With the continued rise of fungicide resistance among major phytopathogens, Myxococcota-derived natural products offer a promising reservoir of novel antifungal compounds. Their metabolic diversity, ecological adaptability, and broad-spectrum activities position them as valuable candidates for next-generation, sustainable crop protection strategies.

## Applications in agriculture: biocontrol against fungal pathogens

### Fungicide resistance: agricultural impact

Several fungal phytopathogens, including *Botrytis cinerea*, *Alternaria alternata*, and *Fusarium* spp., have demonstrated increasing resistance to commonly applied fungicides. In South Africa, antifungal-resistant *B. cinerea* strains have been reported in pear orchards, rooibos nurseries, and vineyards, showing reduced sensitivity to dicarboximides, benzimidazoles, and anilinopyrimidines (Fourie & Holz 1998; Spies 2005; Wessels et al. 2013, 2016). Similarly, *A. alternata* isolated from pecan trees displayed resistance to boscalid, fentin hydroxide, and pyraclostrobin (Achilonu et al. 2023a; 2023b), while *Fusarium* spp. associated with maize and wheat exhibited reduced sensitivity to triazole fungicides (Janse van Rensburg et al. 2016). These findings highlight potential challenges for the long-term efficacy of chemical fungicide-based control strategies.

### Agricultural fungicide resistance and the link with antimicrobial resistance (AMR)

Beyond agricultural implications, fungicide resistance may overlap with clinical antifungal resistance, raising broader public health concerns. The widespread use of azoles in agriculture is linked to resistance in human pathogens including *Cryptococcus neoformans*, *Candida auris*, *Aspergillus fumigatus*, and *Fusarium* spp., with environmental transmission routes playing a key role (Fisher et al. 2022; Bengtsson-Palme et al. 2023; Lockhart et al. 2023; Baker et al. 2024; Williams et al. 2024). Resistant strains emerging in agricultural contexts may disseminate via environmental pathways (soil, water and air), potentially contributing to the global antimicrobial resistance (AMR) burden (Fisher et al. 2022; Davidovich et al. 2025; Sajid et al. 2024). The World Health Organisation (WHO) has recognised antifungal resistance as a major health threat, citing *Cryptococcus*, *Aspergillus*, *Candida*, and *Fusarium* spp. as pathogens of critical concern due to their high mortality rates and limited treatment options (WHO 2022).

The overuse or misapplication of fungicides has been linked to resistance development and may pose environmental and health concerns. Fungicide residues enter soil and water and may disrupt microbial communities and contribute to bioaccumulation, leading to unintended ecological consequences (Wroński et al. 2024). While these challenges may emerge gradually, their cumulative impact warrants proactive monitoring and management. There is, therefore, a growing interest in alternatives that minimise resistance risks while ensuring effective fungal control.

## Myxococcota as a source of antifungal compounds

The increasing resistance to chemical fungicides and associated agricultural and human health concerns have prompted interest in alternative strategies, such as bioprotection. Bioprotection, particularly through biological control (biocontrol) using microbially derived antifungals [non-living, nature-based substances], has emerged as a potential alternative (Stenberg et al. 2021). Due to their natural origin and unique mechanisms of action, Myxococcota-derived secondary metabolites may offer agricultural potential, particularly in South Africa’s agrarian sector (Li et al. 2023; Wang et al. 2024). With rising fungicide resistance rates, Myxococcota-derived secondary metabolites could support long-term, sustainable crop protection and help reduce the dependence on chemical fungicides.

Myxococcota possess large genomes (9 to 14 megabases), with 6 to 10% dedicated to BGCs. The phylum Myxococcota contains ~ 1895 known BGCs (Whitworth et al. 2021; Sourice et al. 2024; Wang et al. 2024). These BGCs encode enzymes involved in PKSs, NRPSs, and hybrid PKS/NRPS systems. These biosynthetic pathways generate bioactive compounds that act on various cellular structures and pathways through multifaceted mechanisms (Bhat et al. 2021; Wang et al. 2024). Myxococcota-derived compounds mediate antifungal activity by interfering with processes including mitochondrial respiration (targeting complexes I and III of the electron transport chain), DNA replication, transcription and translation, cell membrane integrity and osmoregulation pathways, acetyl-CoA carboxylase, and metal ion chelation. The functional specificity of Myxococcota biosynthetic enzymes and the structural diversity of secondary metabolites gives rise to their multifaceted modes of action (Weissman and Müller 2010).

### Antifungal activity of polyketides (PKs)

PK biosynthesis proceeds through stepwise assembly, incorporating short-chain acyl starting substrates and extension units such as acetyl-CoA, propionyl-CoA, malonyl-CoA, and methylmalonyl-CoA. Three PKSs catalyse the stepwise assembly of PKs: type I PKSs, type II PKSs, and type III PKSs. Type I PKSs are either modular or iterative enzymes involved in the biosynthesis of macrolides and other complex PKs. Type II PKSs, in contrast, are iterative multi-enzyme complexes that facilitate the biosynthesis of aromatic and polycyclic PKs, while type III PKSs are homodimeric iterative enzymes facilitating the biosynthesis of structurally diverse PKs (Wang et al. 2024; François et al. 2025).

**Fig. 4:**
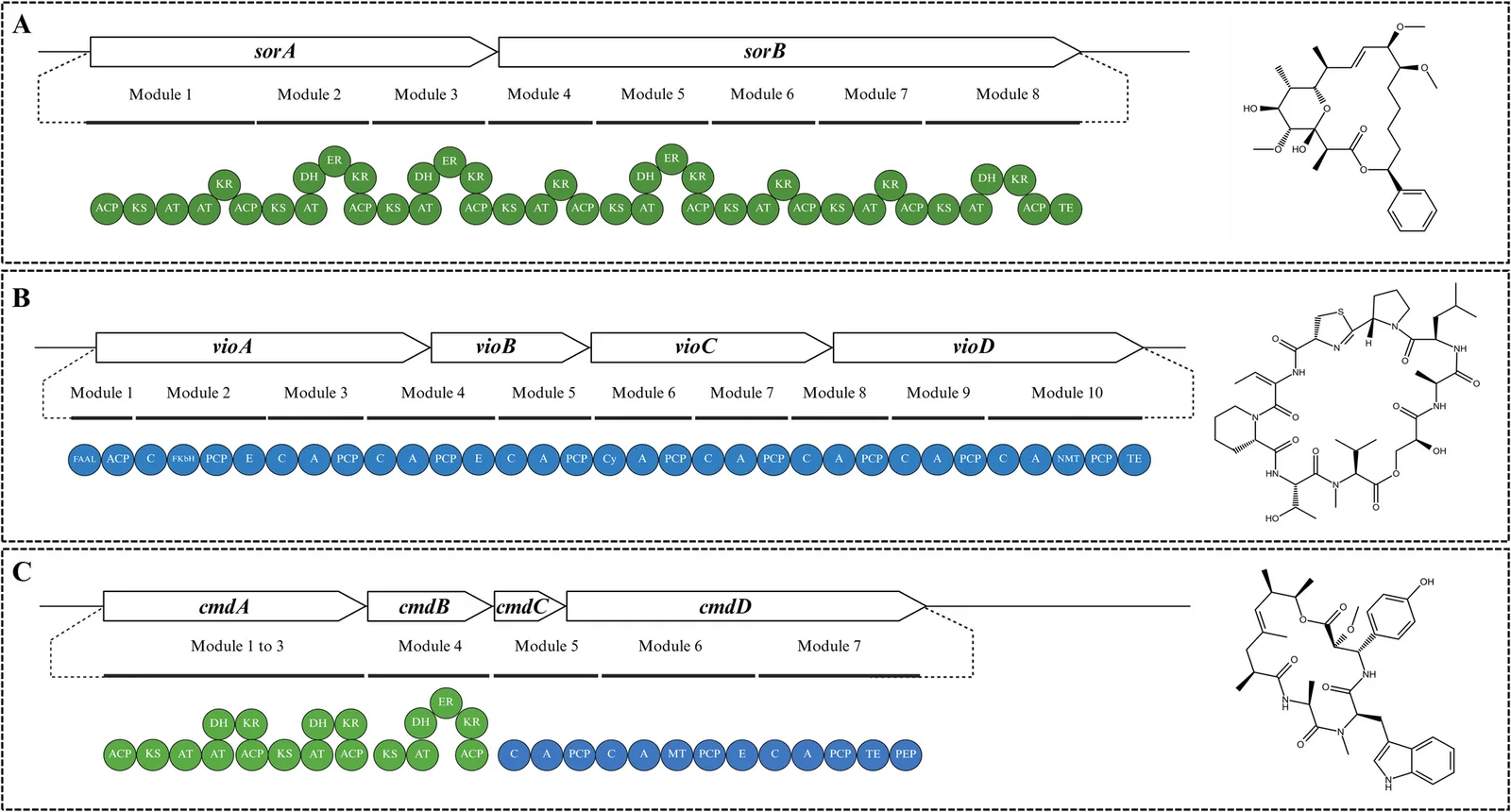
Modular layout of BGCs from Myxococcota involved in the production of secondary metabolites: **A***sorA to sorB* (*Sorangium cellulosum*) encodes a Type I modular polyketide synthase (PKS) system responsible for soraphen A biosynthesis. The modules contain classical PKS domains: ketosynthase (KS), acyltransferase (AT), acyl carrier protein (ACP), β-keto processing enzymes [ketoreductase (KR), dehydratase (DH), enoylreductase (ER)], and a thioesterase (TE) domain for product release; **B***vioA* to *vioD* (*Cystobacter violaceus*) encode a non-ribosomal peptide synthetase (NRPS) system directing vioprolide biosynthesis. This system is composed of condensation (C), adenylation (A), and peptidyl carrier protein (PCP) domains, with an initiating fatty acyl-AMP ligase (FAAL) and FkbH domain for starter unit loading, as well as a terminal thioesterase (TE) domain for peptide release and cyclisation; **C***cmdA to cmdF* (*Chondromyces crocatus*) represents a hybrid PKS/NRPS system responsible for chondramide A biosynthesis. PKS modules (*cmdA* to *cmdB*) contain KS, AT, ACP, and β-keto processing domains, while NRPS modules (*cmdC* to *cmdD*) incorporate C, A, PCP, MT, and E domains. Tailoring genes *cmdE* and *cmdF* encode a halogenase and tyrosine aminomutase, respectively, facilitating post-assembly modifications. Chemical structures of soraphen, vioprolide, and chondramide. Structures were obtained from PubChem (https://pubchem.ncbi.nlm.nih.gov/) and redrawn using the RCSB PDB chemical sketch tool (https://www.rcsb.org/chemical-sketch) (adapted from Zirkle et al. 2004; Rachid et al. 2006; Yan et al. 2018; Created with BioRender.com)

A well-characterised example is soraphen A BGC from *Sorangium cellulosum* (Fig. 4A; Supplementary File 2). A type I PKS system, encoded by two adjacent genes, *sorA* and *sorB*, directs soraphen biosynthesis by assembling eight enzymatic modules with typical PKS domains [acyltransferase (AT), KS, acyl carrier protein (ACP), ketoreductase (KR), dehydratase (DH), enoyl reductase (ER), and thioesterase (TE)] (Ligon et al. 2002; Zirkle et al. 2004). Tailoring enzymes, including SorM (methyltransferase) and SorR (oxidase), along with the sorCDEF operon, modify the PK scaffold and supply methoxymalonyl-CoA building blocks (Zirkle et al. 2004; Naini et al. 2019). Soraphen A has been observed to exhibit antifungal activity against the filamentous fungi *Mucor hiemalis* and *B. cinerea*, as well as the yeasts *Candida albicans* and *Nematospora coryli*^1^ (Gerth et al. 1994) (Supplementary File 2).

In addition to soraphen A, *S. cellulosum* was reported to have produced one of the earliest characterised macrolactones, ambruticin. Ambruticin was reported to exhibit antifungal activity against *Coccidioides immitis* and *Histoplasma capsulatum*, as well as *B. cinerea* and *Alternaria tenuis* (Ringel et al. 1977) (Supplementary File 2). Additionally, *S. cellulosum* strains also produce a variety of other bioactive compounds, including macrolides (disorazol, chivosazol, and epothilone), aromatics (jerangolid), quinones (sorangiolid), and polyenes (ratjadone) (Yue et al. 2023). For example, Gerth et al. (1996a, 1996b) isolated epothilone A and B from *S. cellulosum*, both exhibited antifungal activity against *M. hiemalis* (Supplementary File 2).

Several other Myxococcota genera have been reported to produce bioactive compounds. *Chondromyces crocatus* is known to produce crocacin, which exhibits antifungal activity against several yeasts and filamentous fungal species. Among yeast species, *C. albicans* and *Metschnikowia pulcherrima* displayed susceptibility to crocacin. For filamentous fungi, *B. cinerea*, *Fusarium fujikuroi*, *Trichoderma koningii*, *M. hiemalis*, and *Pythium debaryanum* were inhibited (Kunze et al. 1994) (Supplementary File 2). Stigmatellin, produced by *Stigmatella aurantiaca*, has been shown to exhibit antifungal activity against *C. albicans*, *Debaryomyces hansenii*, *Hansenula anomala*, *Nadsonia fulvescens*, *Saccharomyces cerevisiae*, and *Torulopsis glabrata* and inhibits the filamentous fungi *Paecilomyces variotii*, *Penicillium digitatum*, *Polyporus* sp., *Polystictus* sp., and *Rhizoctonia solani* (Kunze et al. 1984) (Supplementary File 2). In parallel, non-ribosomal peptides (NRPs) represent another structurally diverse class of secondary metabolites that may hold promising antifungal potential.

### Antifungal activity of non-ribosomal peptides (NRPs)

Compared to PKs, NRPS systems synthesise NRPs in a biosynthetic pathway without ribosomal complexes and mRNA templates. In contrast to traditional ribosomal peptide synthesis, NRPSs assemble proteinogenic and non-proteinogenic amino acids to form structurally complex and bioactive peptides. Standard NRPSs contain three core functional domains: adenylation (A), peptidyl carrier protein [PCP or thiolation (T)], and C domains, each involved in key biosynthetic steps including substrate recognition and activation, intermediate transport, and peptide bond formation, respectively (Patil and Gore 2024). In Myxococcota, NRPSs often include additional tailoring domains, such as epimerisation (E), heterocyclisation (Cy), and methyltransferase (MT), which function during peptide elongation or post-synthesis (Groß et al. 2023).

Vioprolides have been identified as NRPs produced by *Cystobacter violaceus* (Schummer et al. 1996; Yan et al. 2018). The biosynthetic genes *vioA* to *vioD*, comprising 10 modules (two unusual and eight standard NRPS modules), are flanked by accessory genes *vioE* to *vioL* (Fig. 4B). These modules contain standard NRPS domains, A, PCP, and C, as well as tailoring domains including a fatty acyl-AMP ligase (FAAL) and an FkbH domain, which facilitate an unusual glycerate esterification process (Auerbach et al. 2018). Vioprolide biosynthesis initiates with fatty acid to ACP and loading of glycerate, followed by ester-bond formation and incorporation of proteinogenic and non-proteinogenic amino acids. The terminal TE domain mediates peptide release and cyclisation. Upstream of the core biosynthetic genes, accessory enzymes such as an ornithine cyclodeaminase-like protein (VioL) contribute to structural diversification and amino acid tailoring (Auerbach et al. 2018; Yan et al. 2018). Although not extensively studied, vioprolide has been reported to possess antifungal activity (Schummer et al. 1996) (Supplementary File 2).

Several pure NRPs, including the argyrin cyclic peptides from *Archangium gephyra*, have been identified in Myxococcota (Sasse et al. 2002). Argyrin variants A to D have been reported to exhibit antifungal activity primarily against filamentous fungi (Supplementary File 2). Argyrin B exhibited inhibitory activity against *B. cinerea* and *P. debaryanum*, comparable to that observed for variants A and C. In contrast, none of the argyrin variants A to D exhibited activity against the yeasts *C. albicans*, *H. anomala*, or *M. pulcherrima*, indicating a selective antifungal spectrum (Sasse et al. 2002).

Many characterised NRPS-derived compounds have demonstrated potent activity against both Gram-positive and Gram-negative bacteria. Representative examples include myxovalargin (*M. fulvus*), cystobactamids (*C. violaceus*), corramycin (*C. coralloides*), and thaxteramides (*Jahnella thaxteri*) (Irschik et al. 1983; Baumann et al. 2014; Oueis et al. 2019; Couturier et al. 2022). While the antifungal activity of NRPS-derived compounds remains understudied, the functional specificity of their biosynthetic enzymes provides a foundation for more intricate hybrid systems, such as PKS/NRPS systems (Weissman & Müller 2010; Baumann et al. 2014; Groß et al. 2023).

### Antifungal activity of hybrid PKS/NRPS systems

While PKS and NRPS biosynthetic pathways exhibit differences in substrate specificity, catalytic mechanisms, and domain organisation, they share similar building blocks, including acyl-CoA derivatives, aryl acids, and proteinogenic and non-proteinogenic amino acids. This overlap has led to the evolution of hybrid PKS/NRPS systems in Myxococcota, facilitating the production of structurally diverse secondary metabolites (Groß et al. 2023).

Chondramides are hybrid PK/NRP metabolites produced by *C. crocatus*, synthesised through a PKS/NRPS system encoded by the *cmdA* to *cmdF* genes (Fig. 4C; Supplementary File 2). This BGC comprises six core genes, organised in a linear order, from *cmdA* to *cmdF*. The genes *cmdA* and *cmdB* encode PKSs, while the *cmdC* and *cmdD* encode NRPSs. The *cmdA* and *cmdB* genes initiate biosynthesis, encoding type I PKS modules that contain KS, AT, DH, KR, and ACP domains for PK synthesis using methylmalonyl-CoA. The NRPS modules extend the intermediate using C, A, and PCP domains, along with tailoring domains such as MT and E, which enable N-methylation and incorporation of D-amino acids. The TE domain in the final NRPS module facilitates product release and cyclisation. Additionally, *cmdE* encodes a halogenase required for chondramide biosynthesis that introduces halogens into specific variants (B and D), and *cmdF*, which produces β-tyrosine through a tyrosine aminomutase (Rachid et al. 2006).

Kunze et al. (1995) demonstrated the antifungal activity of chondramide variants produced by *C. crocatus*. Chondramide B showed antifungal activity against strains of *C. albicans*, *H. anomala*, *Lipomyces lipofer*, and *T. glabrata*, while chondramide C was active against *Schizosaccharomyces pombe*. In contrast, chondramide D showed the highest antifungal activity against *C. albicans*. Additionally, Kunze et al. (2008) isolated and characterised pedein A and B from *Chondromyces pediculatus*, which exhibited antifungal activity against *C. albicans*, *S. cerevisiae*, *Rhizopus arrhizus*, *T. koningii*, and *B. cinerea*. In comparison, archazolid A produced by *A. gephyra* exhibited a broader antifungal spectrum, inhibiting yeast including *H. anomala*, *M. pulcherrima*, *Pichia membranifaciens*, *S. cerevisiae*, *T. glabrata*, and filamentous fungi such as *Aspergillus niger*, *B. cinerea*, *M. hiemalis*, *P. debaryanum*, and *Ustilago zeae* (Sasse et al. 2003) (Supplementary File 2).

In contrast, miuraenamide A, produced by *Paraliomyxa miuraensis*, represents an example of targeted antifungal activity, exhibiting high potency against *Phytophthora capsici*, *Rhizopus oryzae*, *Absidia spinosa*, and *Trichophyton mentagrophytes*. However, miuraenamide A was reported to be ineffective against *A. niger* and *B. cinerea*. Among yeast species, *Candida rugosa* and *Rhodotorula minuta* (*Cystobasidium minutum*) were most susceptible, followed by *Pichia burtonii*, whereas *S. cerevisiae* exhibited reduced susceptibility (Iizuka et al. 2006) (Supplementary File 2). More recently, *Citreicoccus inhibens* was reported to produce aminopyrrolnitrin and pyrrolnitrin, exhibiting antifungal activity against *Fusarium oxysporum* (Zhou et al. 2021).

Similarly, microsclerodermins, originally isolated from marine sponges and later detected in Myxococcota, exhibit targeted antifungal activity. Bewley et al. (1994) first described microsclerodermins A and B as antifungal cyclic peptides capable of inhibiting *C. albicans*. Subsequently, Hoffmann et al. (2013) identified additional analogues with similar bioactivity, including microsclerodermins M, L, and D, all of which exhibited potential antifungal activity against *C. albicans* (Supplementary File 2). Additionally, myxalamid, produced by *Myxococcus xanthus*, has shown selective antifungal activity against *Paecilomyces* sp., *Mucor lusitanicus*, *Rhodosporidium glutinis*, and *N. fulvescens*. However, in the study, myxalamid did not exhibit activity against *M. hiemalis*, *N. coryli*, *C. albicans*, *T. glabrata*, *H. anomala*, *P. membranifaciens*, *D. hansenii*, *S. cerevisiae*, and *S. pombe* (Gerth et al. 1983). In contrast, aetheramides, a distinct class of cyclic NRPs produced by *Aetherobacter rufus*, have also been reported to possess antifungal activity against *C. albicans* (Plaza et al. 2012). Beyond these examples, additional Myxococcota-derived PKs, NRPs, and hybrid PK/NRPs further expand the antifungal array (Supplementary File2). This biosynthetic diversity has led to the investigation of Myxococcota as a promising biocontrol strategy in agricultural settings (Dahm et al. 2015; Ye et al. 2020; Wu et al. 2021; Xia et al. 2023; Han et al. 2025).

## International case studies: Myxococcota in agricultural applications

To date, no published studies have investigated the application of Myxococcota in South African agriculture; however, international case studies offer valuable insights into their potential. Several international studies have demonstrated their antifungal potential in diverse cropping systems (Dahm et al. 2015; Wu et al. 2021; Xia et al. 2023). These studies provide preliminary mechanistic insights into Myxococcota host–pathogen interactions and serve as proof-of-concept models. These findings underscore a notable research gap in the South African context and point to the need for local bioprospecting and functional validation of endemic strains. A synthesis of global findings may help inform future research and application within South Africa’s distinct agro-ecological settings.

Several international studies have explored the potential of Myxococcota as a biological control agent in agricultural systems (Bhat et al. 2021). For example, Dahm et al. (2015) reported biocontrol activity of *Myxococcus virescens* and *Corallococcus exiguus* against fungal pathogens (*R. solani*, *F. oxysporum*, and *Cylindrocarpon destructans*) of forest trees. These Myxococcota species suppressed fungal growth by 38% to 63%, while in vivo tests on *Pinus sylvestris* seedlings showed protective effects against *R. solani*. A 2-year study by Ye et al. (2020) demonstrated that *Corallococcus* sp. strain EGB reduced *F. oxysporum* f. sp. *cucumerinum* (*Fusarium* wilt) by 79.6% (greenhouse) and 53.9 to 66.0% (field). Additionally, metagenomic analysis of the soil revealed microbial restructuring, characterised by reduced diversity and increased connectivity, which may suggest competition and predation following the introduction of *Corallococcus* sp. strain EGB.

Similarly, Wu et al. (2021) investigated the biocontrol potential of *M. xanthus* B25-I-1 against *Phytophthora infestans* (potato late blight). The study demonstrated that *M. xanthus* B25-I-1 inhibited *P. infestans* mycelial growth by 45.45% at 10 μg/mL and completely at 1000 μg/mL. Detached potato leaf assays further validated the biocontrol potential, as the active extract significantly reduced *P. infestans* infection without harming plant tissues. Additionally, Xia et al. (2023) demonstrated that *Corallococcus* sp. EGB effectively controls *Phytophthora sojae* (soybean root rot) by secreting a thiaminase enzyme (CcThi1). In greenhouse trials, *Corallococcus* sp. EGB significantly reduced soybean root rot by 50.34% compared to 46.23% by *M. xanthus* DK1622. More recently, Han et al. (2025) assessed the biocontrol potential of *Cystobacter fuscus* HM-E against *Verticillium dahliae* (cotton *Verticillium* wilt). *Cystobacter fuscus* HM-E was observed to inhibit spore germination (83.5%) significantly and resulted in spore lysis (71.7%) of *V. dahliae*. Additionally, greenhouse trials further demonstrated the biocontrol potential of *C. fuscus* HM-E, where the application of fermentation broth resulted in 23.01% disease control. In comparison, a solid formulation improved efficacy to 70.90%.

While these studies demonstrate the potential biocontrol efficacy of Myxococcota in several crops, studies on wheat and maize remain limited. Future studies may focus on isolating Myxococcota from endemic South African biomes and evaluating their activity against phytopathogenic fungi of wheat, maize, and other crops where fungicide resistance is emerging.

## Outlook and Future Directions

South Africa’s diverse and underexplored biomes, ranging from nutrient-poor Fynbos soils to arid deserts and biologically rich savannas offer a promising frontier for bioprospecting. These environments may harbour novel Myxococcota and untapped BGCs with biotechnological relevance. Systematic sampling, supported by strain repositories, genomics, and screening, is essential to realise this potential.

Although genomics and metagenomics have significantly advanced, several limitations still prevent the full realisation of microbial biosynthetic potential. MAGs assembled from short-read sequencing data lack continuity, particularly in regions encoding large modular enzymes such as PKSs and NRPSs. Moreover, the underrepresentation of uncultured taxa, including Myxococcota, in existing reference databases limits functional annotation and metabolite prediction, leaving much of the microbial secondary metabolism unexplored. Future efforts should combine hybrid sequencing and comparative genomics to improve BGC recovery and classification. In parallel, computational predictions should be validated experimentally. Comprehensive platforms should, therefore, be developed that encompass high-throughput isolation and screening, hybrid genomic (Illumina MiSeq with PacBio or ONT) and metabolomic profiling, and heterologous expression, enabling direct connections between BGCs and their corresponding bioactivities.

Considering the increasing resistance to chemical fungicides and associated agricultural and human health concerns, Myxococcota-derived secondary metabolites may represent promising bioprotective candidates for sustainable crop protection. Achieving this potential will depend on interdisciplinary collaboration across the life sciences, with microbial natural products offering a promising route towards AMR mitigation and sustainable agriculture.

## Conclusion

Myxococcota are ecologically versatile bacteria characterised by predatory behaviour and prolific production of secondary metabolites. However, Myxococcota remain understudied within South Africa’s ecologically diverse biomes. This underrepresentation is due to limitations of conventional culture-based isolation methods, which are constrained by the slow growth of Myxococcota, predatory nature, complex community interactions, and sensitivity to laboratory conditions. While MAGs and genome mining offer bioinformatic tools to explore the biosynthetic potential of uncultured or low-abundance Myxococcota, these methods remain constrained by incomplete assemblies of modular BGCs, dependence on reference databases, and the lack of functional validation through heterologous expression. Nevertheless, numerous secondary metabolites have been identified in global studies to date, including myxothiazol, ambruticin, crocacin, chondramides, and miuraenamide, which exhibit antifungal activity against fungal phytopathogens, highlighting their agricultural relevance.

While international studies have documented the bioactivity of Myxococcota-derived secondary metabolites, their potential for bioprotection in South African agriculture remains largely understudied. The growing resistance to chemical fungicides further highlights the need for systematic bioprospecting. Future research should prioritise integrative approaches that combine conventional culture-based methods with hybrid high-throughput genomic analyses and in vitro functional validation. South Africa’s ecologically diverse biomes may represent untapped reservoirs for future exploration of Myxococcota with the potential to support sustainable agriculture, expand microbial ecological understanding, and uncover novel natural products. Addressing these research gaps will help establish the position of South African Myxococcota as an untapped resource for microbial ecology and biotechnology.

## Supplementary Information

Below is the link to the electronic supplementary material.

Supplementary Information 1 (XLSX 17.5 KB)

Supplementary Information 2 (XLSX 28.1 KB)

Supplementary Information 3 (DOCX 32.3 KB)

## Data Availability

No datasets were generated or analysed during the current study.

## Footnotes

1 Scientific names are presented as reported in the original sources to maintain historical accuracy.
