# Supplementary material for: South African Myxococcota: an untapped resource for microbial ecolo gy and biotechnology
Source: Appl Microbiol Biotechnol. 2025 Aug 30;109(1):192. doi: 10.1007/s00253-025-13586-z (PMC12397197; doi:10.1007/s00253-025-13586-z)
Supplement: Supplementary file 3 — Supplementary Information 3 (DOCX 32.3 KB) [file 253_2025_13586_MOESM3_ESM.docx]

**Applied Microbiology and Biotechnology: Supplementary Information 3**

South African Myxococcota: An Untapped Resource for Microbial Ecology and Biotechnology

**Authors**

Benjamin Havenga^1^ [0000-0002-7634-0605]

Karin Jacobs^1^ [0000-0003-3972-5343]

**Affiliation**

^1^Department of Microbiology, Faculty of Science, Stellenbosch University, Stellenbosch 7602, South Africa

**Corresponding author**

Benjamin Havenga (bhavenga@sun.ac.za)

# **Supplementary References**

Barbier J, Jansen R, Irschik H, Benson S, Gerth K, Böhlendorf B, Höfle G, Reichenbach H, Wegner J, Zeilinger C, Kirschning A, Müller R (2012) Isolation and total synthesis of icumazoles and noricumazoles – Antifungal antibiotics and cation‐channel blockers from *Sorangium cellulosum*. Angew Chem Int Ed 51(5):1256–1260. https://doi.org/10.1002/anie.201106435

Bernard Dupont (2014) S37 Road North of Tshokwane, Kruger National Park, South Africa. Wikimedia Commons. https://commons.wikimedia.org/wiki/File:Green_Savanna_..._(13645333993).jpg. Accessed 22 April 2025

Bernard Dupont (2023) Addo Elephant National Park, Eastern Cape, South Africa. Wikimedia Commons. https://commons.wikimedia.org/wiki/File:Lush_Valley_at_Addo_Elephant_National_Park.jpg. Accessed 22 April 2025

Bhat MA, Mishra AK, Bhat MA, Banday MI, Bashir O, Rather IA, Rahman S, Shah AA, Jan AT (2021) Myxobacteria as a source of new bioactive compounds: a perspective study. Pharmaceutics 13(8):1265. https://doi.org/10.3390/pharmaceutics13081265

Bode HB, Irschik H, Wenzel SC, Reichenbach H, Müller R, Höfle G (2003) The leupyrrins: a structurally unique family of secondary metabolites from the myxobacterium *Sorangium cellulosum*. J Nat Prod 66(9):1203–1206. https://doi.org/10.1021/np030109v

Brasseur G, Di Rago JP, Slonimski PP, Lemesle-Meunier D (2001) Analysis of suppressor mutation reveals long distance interactions in the *bc*_1_ complex of *Saccharomyces cerevisiae*. Biochim Biophys Acta Bioenerg 1506(2):89–102. https://doi.org/10.1016/s0005-2728(01)00186-4

Darren Glanville (2018) Hluhluwe-iMfolozi Park. Wikimedia Commons. https://commons.wikimedia.org/wiki/File:Hluhluwe%E2%80%93iMfolozi_Park_-_30226064977.jpg. Accessed 22 April 2025

Francesco Bandarin (2005) iSimangaliso Wetland Park (South Africa). Wikimedia Commons. https://commons.wikimedia.org/wiki/File:ISimangaliso_Wetland_Park-113322.jpg. Accessed 22 April 2025

Fudou R, Iizuka T, Sato S, Ando T, Shimba N, Yamanaka S (2001a) Haliangicin, a novel antifungal metabolite produced by a marine myxobacterium. Isolation and structural elucidation. J Antibiot 54(2):153–156. https://doi.org/10.7164/antibiotics.54.153

Fudou R, Iizuka T, Yamanaka S (2001b) Haliangicin, a novel antifungal metabolite produced by a marine myxobacterium. Fermentation and biological characteristics. J Antibiot 54(2):149–52. https://doi.org/10.7164/antibiotics.54.149

Gerth K, Bedorf N, Höfle G, Irschik H, Reichenbach H (1996b) Epothilons A and B: antifungal and cytotoxic compounds from Sorangium cellulosum (myxobacteria). Production, physico-chemical and biological properties. J Antibiot 49(6):560–3. https://doi.org/10.7164/antibiotics.49.560

Gerth K, Bedorf N, Irschik H, Höfle G, Reichenbach H (1994) The soraphens: a family of novel antifungal compounds from *Sorangium cellulosum* (myxobacteria). Soraphen A 1α: Fermentation, isolation, biological properties. J Antibiot 47(1):23–31. https://doi.org/10.7164/antibiotics.47.23

Gerth K, Jansen R, Reifenstahl G, Höfle G, Irschik H, Kunze B, Reichenbach H, Thierbach G (1983). The myxalamids, new antibiotics from *Myxococcus xanthus* (myxobacterales). Production, physico-chemical and biological properties, and mechanism of action. J Antibiot 36(9):1150–6. https://doi.org/10.7164/antibiotics.36.1150

Gerth K, Schummer D, Höfle G, Irschik H, Reichenbach H (1995) Ratjadon: a new antifungal compound from Sorangium cellulosum (myxobacteria) production, physico-chemical and biological properties. J Antibiot 48(9):973–6. https://doi.org/10.7164/antibiotics.48.973

Gerth K, Trowitzsch W, Wray V, Höfle G, Irschik H, Reichenbach H (1982) Pyrrolnitrin from *Myxococcus fulvus* (myxobacterales). J Antibiot 35(8):1101–3. https://doi.org/10.7164/antibiotics.35.1101

Gerth K, Washausen P, Höfle G, Irschik H, Reichenbach H (1996a) The Jerangolids: a family of new antifungal compounds from *Sorangium cellulosum* (myxobacteria). Production, physico-chemical and biological properties of Jerangolid A. J Antibiot 49(1):71–75. https://doi.org/10.7164/antibiotics.49.71

Gulder TAM, Neff S, Schüz T, Winkler T, Gees R, Böhlendorf B (2013) The myxocoumarins A and B from *Stigmatella aurantiaca* strain MYX-030. Beilstein J Org Chem 9:2579–2585. https://doi.org/10.3762/bjoc.9.293

Hoffmann H, Kogler H, Heyse W, Matter H, Caspers M, Schummer D, Klemke‐Jahn C, Bauer A, Penarier G, Debussche L, Brönstrup M (2015) Discovery, structure elucidation, and biological characterization of nannocystin A, a macrocyclic myxobacterial metabolite with potent antiproliferative properties. Angew Chem Int Ed 54(35):10145–10148. https://doi.org/10.1002/anie.201411377

Hoffmann T, Müller S, Nadmid S, Garcia R, Müller R (2013) Microsclerodermins from terrestrial myxobacteria: an intriguing biosynthesis likely connected to a sponge symbiont. J Am Chem Soc 135(45):16904–16911. https://doi.org/10.1021/ja4054509

Iizuka T, Fudou R, Jojima Y, Ogawa S, Yamanaka S, Inukai Y, Ojika M (2006) Miuraenamides A and B, novel antimicrobial cyclic depsipeptides from a new slightly halophilic myxobacterium: taxonomy, production, and biological properties. J Antibiot 59(7):385–391. https://doi.org/10.1038/ja.2006.55

Irschik H, Augustiniak H, Gerth K, Höfle G, Reichenbach H (1995c) The ripostatins, novel inhibitors of eubacterial RNA polymerase isolated from myxobacteria. J Antibiot 48(8):787–792. https://doi.org/10.7164/antibiotics.48.787

Irschik H, Jansen R, Gerth K, Höfle G, Reichenbach H (1995a) Disorazol A, an efficient inhibitor of eukaryotic organisms isolated from myxobacteria. J Antibiot 48(1):31–35. https://doi.org/10.7164/antibiotics.48.31

Irschik H, Jansen R, Gerth K, Höfle G, Reichenbach H (1995b) Chivosazol A, a new inhibitor of eukaryotic organisms isolated from myxobacteria. J Antibiot 48(9):962–966. https://doi.org/10.7164/antibiotics.48.962

Jansen R, Kunze B, Reichenbach H, Höfle G (2003) Chondrochloren A and B, new β‐amino styrenes from *Chondromyces crocatus* (myxobacteria). Eur J Org Chem 2003(14):2684–2689. https://doi.org/10.1002/ejoc.200200699

Jansen R, Mohr KI, Bernecker S, Stadler M, Müller R (2014a) Indothiazinone, an indolyl thiazolyl ketone from a novel myxobacterium belonging to the Sorangiineae. J Nat Prod 77(4):1054–1060. https://doi.org/10.1021/np500144t

Jansen R, Sood S, Huch V, Kunze B, Stadler M, Müller R (2014b) Pyrronazols, metabolites from the myxobacteria *Nannocystis pusilla* and *N. exedens*, are unusual chlorinated pyrone-oxazole-pyrroles. J Nat Prod 77(2):320–326. https://doi.org/10.1021/np400877r

Jansen R, Sood S, Mohr KI, Kunze B, Irschik H, Stadler M, Müller R (2014c) Nannozinones and sorazinones, unprecedented pyrazinones from myxobacteria. J Nat Prod 77(11):2545–2552. https://doi.org/10.1021/np500632c

Jansen R, Washausen P, Kunze B, Reichenbach H, Höfle G (1999) The crocacins, novel antifungal and cytotoxic antibiotics from *Chondromyces crocatus* and *Chondromyces pediculatus* (myxobacteria): isolation and structure elucidation. Eur J Org Chem 30(30):1085–1089. https://doi.org/10.1002/chin.199930200

Kim YJ, Furihata K, Yamanaka S, Fudo R, Seto H (1991) Isolation and structural elucidation of stipiamide, a new antibiotic effective to multidrug-resistant cancer cells. J Antibiot 44(5):553–556. https://doi.org/10.7164/antibiotics.44.553

Krastel P, Roggo S, Schirle M, Ross NT, Perruccio F, Aspesi P Jr, Aust T, Buntin K, Estoppey D, Liechty B, Mapa F, Memmert K, Miller H, Pan X, Riedl R, Thibaut C, Thomas J, Wagner T, Weber E, Xie X, Schmitt EK, Hoepfner D (2015) Nannocystin A: an elongation factor 1 inhibitor from myxobacteria with differential anti‐cancer properties. Angew Chem Int Ed 54(35):10149–10154. https://doi.org/10.1002/anie.201505069

Kunze B, Böhlendorf B, Reichenbach H, Höfle G (2008) Pedein A and B: production, isolation, structure elucidation and biological properties of new antifungal cyclopeptides from *Chondromyces pediculatus* (myxobacteria). J Antibiot 61(1):18–26. https://doi.org/10.1038/ja.2008.104

Kunze B, Höfle G, Reichenbach H (1987) The aurachins, new quinoline antibiotics from myxobacteria: Production, physico-chemical and biological properties. J Antibiot 40(3):258–265. https://doi.org/10.7164/antibiotics.40.258

Kunze B, Jansen R, Höfle G, Reichenbach H (1994) Crocacin, a new electron transport inhibitor from Chondromyces crocatus (myxobacteria): production, isolation, physico-chemical and biological properties. J Antibiot 47(8):881–886. https://doi.org/10.7164/antibiotics.47.881

Kunze B, Jansen R, Höfle G, Reichenbach H (2004) Ajudazols, new inhibitors of the mitochondrial electron transport from *Chondromyces crocatus*: production, antimicrobial activity and mechanism of action. J Antibiot 57(2):151–155. https://doi.org/10.7164/antibiotics.57.151

Kunze B, Jansen R, Pridzun L, Jurkiewicz E, Hunsmann G, Höfle G, Reichenbach H (1992b) Phenoxan, a new oxazole-pyrone from myxobacteria: production, antimicrobial activity and its inhibition of the electron transport in complex I of the respiratory chain. J Antibiot 45(9):1549–1552. https://doi.org/10.7164/antibiotics.45.1549

Kunze B, Jansen R, Pridzun L, Jurkiewicz E, Hunsmann G, Höfle G, Reichenbach H (1993) Thiangazole, a new thiazoline antibiotic from *Polyangium* sp. (myxobacteria): production, antimicrobial activity and mechanism of action. J Antibiot 46(11):1752–1755. https://doi.org/10.7164/antibiotics.46.1752

Kunze B, Jansen R, Sasse F, Höfle G, Reichenbach H (1995) Chondramides A–D, new antifungal and cytostatic depsipeptides from *Chondromyces crocatus* (myxobacteria): production, physico-chemical and biological properties. J Antibiot 48(11):1262–1266. https://doi.org/10.7164/antibiotics.48.1262

Kunze B, Kemmer T, Höfle G, Reichenbach H (1984) Stigmatellin, a new antibiotic from *Stigmatella aurantiaca* (myxobacterales). Production, physico-chemical and biological properties. J Antibiot 37(5):454–461. https://doi.org/10.7164/antibiotics.37.454

Kunze B, Reichenbach H, Müller R, Höfle G (2005) Aurafuron A and B, new bioactive polyketides from *Stigmatella aurantiaca* and *Archangium gephyra* (myxobacteria). J Antibiot 58(4):244–251. https://doi.org/10.1038/ja.2005.28

Kunze B, Steinmetz H, Höfle G, Huss M, Wieczorek H, Reichenbach H (2006) Cruentaren, a new antifungal salicylate-type macrolide from *Byssovorax cruenta* (myxobacteria) with inhibitory effect on mitochondrial ATPase activity: fermentation and biological properties. J Antibiot 59(10):664–668. https://doi.org/10.1038/ja.2006.89

Kunze B, Trowitzsch-Kienast W, Höfle G, Reichenbach H (1992a) Nannochelins A, B and C, new iron-chelating compounds from *Nannocystis exedens* (myxobacteria): production, isolation, physico-chemical and biological properties. J Antibiot 45(2):147–150. https://doi.org/10.7164/antibiotics.45.147

Mohr KI, Garcia RO, Gerth K, Irschik H, Müller R (2012) *Sandaracinus amylolyticus* gen. nov., sp. nov., a starch-degrading soil myxobacterium, and description of *Sandaracinaceae* fam. nov. Int J Syst Evol Microbiol 62(5):1191–1198. https://doi.org/10.1099/ijs.0.033696-0

Niggemann J, Bedorf N, Flörke U, Steinmetz H, Gerth K, Reichenbach H, Höfle G (2005) Spirangien A and B, highly cytotoxic and antifungal spiroketals from the myxobacterium *Sorangium cellulosum*: isolation, structure elucidation and chemical modifications. Eur J Org Chem 2005(23):5013–5018. https://doi.org/10.1002/ejoc.200500425

Ojika M, Suzuki Y, Tsukamoto A, Sakagami Y, Fudou R, Yoshimura T, Yamanaka S (1998) Cystothiazoles A and B, new bithiazole-type antibiotics from the myxobacterium *Cystobacter fuscus*. J Antibiot 51(3):275–281. https://doi.org/10.7164/antibiotics.51.275

Okanya PW, Mohr KI, Gerth K, Kessler W, Jansen R, Stadler M, Müller R (2014) Hyafurones, hyapyrrolines, and hyapyrones: polyketides from *Hyalangium minutum.* J Nat Prod 77(6):1420–1429. https://doi.org/10.1021/np500145f

Okanya PW, Mohr KI, Gerth K, Steinmetz H, Huch V, Jansen R, Müller R (2012) Hyaladione, an S-methyl cyclohexadiene-dione from *Hyalangium minutum*. J Nat Prod 75(4):768–770. https://doi.org/10.1021/np200776v

Pavel Špindler (2014) Golden Gate Highlands National Park, South Africa. Wikimedia Commons. https://commons.wikimedia.org/wiki/File:Golden_Gate_Highlands_National_Park,_South_Africa_-_panoramio_(3).jpg. Accessed 22 April 2025

Perlova O, Fu J, Kuhlmann S, Krug D, Stewart AF, Zhang Y, Müller R (2006) Reconstitution of the myxothiazol biosynthetic gene cluster by Red/ET recombination and heterologous expression in *Myxococcus xanthus*. Appl Environ Microbiol 72(12):7485–7494. https://doi.org/10.1128/aem.01503-06

Plaza A, Garcia R, Bifulco G, Martinez JP, Hüttel S, Sasse F, Meyerhans A, Stadler M, Müller R (2012) Aetheramides A and B, potent HIV-inhibitory depsipeptides from a myxobacterium of the new genus “*Aetherobacter*” Org Lett 14(11):2854–2857. https://doi.org/10.1021/ol3011002

Popoff A, Hug JJ, Walesch S, Garcia R, Keller L, Müller R (2021) Structure and biosynthesis of myxofacyclines: unique myxobacterial polyketides featuring varing and rare heterocycles. Chem Eur J 27(67):16654–16661. https://doi.org/10.1002/chem.202103095

Ringel SM, Greenough RC, Roemer S, Connor D, Gutt AL, Blair B, Kanter G, Von Strandtmann M (1977) Ambruticin (W7783), a new antifungal antibiotic. J Antibiot 30(5):371–375. https://doi.org/10.7164/antibiotics.30.371

S Molteno (2010) Peninsula Sandstone Fynbos. Cape Town, South Africa. Wikimedia Commons. https://commons.wikimedia.org/wiki/File:Peninsula_Sandstone_Fynbos_-_Cape_Town_8.JPG. Accessed 22 April 2025

Saggu SK, Nath A, Kumar S (2023) Myxobacteria: biology and bioactive secondary metabolites. Res Microbiol 174(7):104079. https://doi.org/10.1016/j.resmic.2023.104079

SAplants (2002) *Romulea tortuosa* in natural habitat; Hantamsberg, Northern Cape. Wikimedia Commons. https://commons.wikimedia.org/wiki/File:Romulea_tortuosa_S-3047.jpg. Accessed 22 April 2025

Sasse F, Böhlendorf B, Hermann M, Kunze B, Forche E, Steinmetz H, Höfle G, Reichenbach H (1999) Melithiazols, new β-methoxyacrylate inhibitors of the respiratory chain isolated from myxobacteria: production, isolation, physico-chemical and biological properties. J Antibiot 52(8):721–729. https://doi.org/10.7164/antibiotics.52.721

Sasse F, Leibold T, Kunze B, Höfle G, Reichenbach H (2003a) Cyrmenins, new β-methoxyacrylate inhibitors of the electron transport: production, isolation, physico-chemical and biological properties. J Antibiot 56(10):827–831. https://doi.org/10.7164/antibiotics.56.827

Sasse F, Steinmetz H, Höfle G, Reichenbach H (1993) Rhizopodin, a new compound from *Myxococcus stipitatus* (myxobacteria) causes formation of rhizopodia-like structures in animal cell cultures: production, isolation, physico-chemical and biological properties. J Antibiot 46(5):741–748. https://doi.org/10.7164/antibiotics.46.741

Sasse F, Steinmetz H, Höfle G, Reichenbach H (1995) Gephyronic acid, a novel inhibitor of eukaryotic protein synthesis from *Archangium gephyra* (myxobacteria): production, isolation, physico-chemical and biological properties, and mechanism of action. J Antibiot 48(1):21–25. https://doi.org/10.7164/antibiotics.48.21

Sasse F, Steinmetz H, Höfle G, Reichenbach H (2003b) Archazolids, new cytotoxic macrolactones from *Archangium gephyra* (myxobacteria): production, isolation, physico-chemical and biological properties. J Antibiot 56(6):520–525. https://doi.org/10.7164/antibiotics.56.520

Sasse F, Steinmetz H, Schupp T, Petersen F, Memmert K, Hofmann H, Heusser C, Brinkmann V, von Matt P, Höfle G, Reichenbach H (2002) Argyrins, immunosuppressive cyclic peptides from myxobacteria. Production, isolation, physico-chemical and biological properties. J Antibiot 55(6):543–551. https://doi.org/10.7164/antibiotics.55.543

Schieferdecker S, Exner TE, Gross H, Roth M, Nett M (2014) New myxothiazols from the predatory bacterium *Myxococcus fulvus*. J Antibiot 67(7):519–525. https://doi.org/10.1038/ja.2014.31

Schummer D, Höfle G, Forche E, Reichenbach H, Wray V, Domke T (1996) Antibiotics from gliding bacteria, Vioprolides: new antifungal and cytotoxic peptolides from Cystobacter violaceus. Liebigs Annalen (6):971–978. https://doi.org/10.1002/jlac.199619960617

South African Tourism (2009) Knysna Forest – Garden Route, South Africa. Wikimedia Commons. https://commons.wikimedia.org/wiki/File:Knysna_Forest_-_Garden_Route,_South_Africa_(3919268880).jpg. Accessed 22 April 2025

South African Tourism (2015) Kalahari Desert, Northern Cape, South Africa. Wikimedia Commons. https://commons.wikimedia.org/wiki/File:Kalahari_desert,_Kalahari,_Northern_Cape,_South_Africa_(19920041014).jpg. Accessed 22 April 2025

Steinmetz H, Mohr KI, Zander W, Jansen R, Gerth K, Müller R (2012) Indiacens A and B: prenyl indoles from the myxobacterium *Sandaracinus amylolyticus*. J Nat Prod 75(10), 1803–1805. https://doi.org/10.1021/np300288b

Thierbach G, Reichenbach H (1981) Myxothiazol, a new inhibitor of the cytochrome *b-c*_1_ segment of the respiratory chain. Biochim Biophys Acta Bioenerg 638(2):282–289. https://doi.org/10.1016/0005-2728(81)90238-3

Wang CY, Hu JQ, Wang DG, Li YZ, Wu C (2024) Recent advances in discovery and biosynthesis of natural products from myxobacteria: an overview from 2017 to 2023. Nat Prod Rep 41(6):905–934. https://doi.org/10.1039/d3np00062a

Wenzel SC, Kunze B, Höfle G, Silakowski B, Scharfe M, Blöcker H, Müller R (2005) Structure and biosynthesis of myxochromides S1–3 in *Stigmatella aurantiaca*: evidence for an iterative bacterial type I polyketide synthase and for module skipping in nonribosomal peptide biosynthesis. ChemBioChem 6(2):375–385. https://doi.org/10.1002/cbic.200400282

Wenzel SC, Meiser P, Binz TM, Mahmud T, Müller R (2006) Nonribosomal peptide biosynthesis: point mutations and module skipping lead to chemical diversity. Angew Chem Int Ed 45(14):2296–2301. https://doi.org/10.1002/anie.200503737

Winfried Bruenken (2001) Namaqualand, Goegap Nature Reserve, Northern Cape, South Africa. Wikimedia Commons. https://commons.wikimedia.org/wiki/File:Namaqualand,_Goegap_0075.jpg. Accessed 22 April 2025
